# A Cross-Sectional Analysis of Body Mass Index With Cholesterol Gallstones in Patients With Cholelithiasis in Tertiary Healthcare Settings

**DOI:** 10.7759/cureus.96214

**Published:** 2025-11-06

**Authors:** Uzma Shamim Seth, Fatima Iqbal, Muhammad Bilal Akhtar, Mustafa Naveed, Rabia Akhtar, Muiz Uddin, Ayesha Shaheen

**Affiliations:** 1 General Surgery, Jinnah Postgraduate Medical Centre, Karachi, PAK; 2 General Surgery, Jinnah Medical and Dental College, Karachi, PAK

**Keywords:** body mass index, cholelithiasis, cholesterol, gallstones, surgery

## Abstract

Background

Gallstones represent a common hepatobiliary disease, with the most common being cholesterol stones. This study aimed to determine the prevalence of cholesterol gallstones and evaluate their relationship with body mass index (BMI) and other risk factors.

Methodology

From March to August 2025, a total of 300 adult patients with ultrasonographically proven cholelithiasis were admitted to the Department of Surgery of Jinnah Medical and Dental College in Karachi and participated in this descriptive cross-sectional study. Patients with hemolytic disorders, chronic liver disease, acute cholecystitis, choledocholithiasis, or previous biliary surgery were not included. Demographic, lifestyle, family history, and clinical data were collected. Weight and height measurements were used to calculate BMI. Gallstones were categorized as mixed, pigment, and cholesterol. Data were analyzed using SPSS version 26 (IBM Corp., Armonk, NY, USA), and chi-square tests were used to evaluate associations (p < 0.05).

Results

A higher BMI was significantly correlated with the presence of cholesterol stones, which were found in 160 (53.3%) patients (p < 0.001). Women comprised the majority (n = 261, 87%) and were more likely to have a high BMI and cholesterol stones. Lifestyle factors and family history also played a role.

Conclusions

There was a strong correlation between elevated BMI and cholesterol gallstones, especially in women. For management and prevention, controlling weight and addressing modifiable risk factors are essential.

## Introduction

Gallstone disease (cholelithiasis) remains the most common disorder of the hepatobiliary system worldwide. The geographic prevalence varies within the wide range of 3% to 21.9% [1]. The global health impact of gallstones is considerable, as the complications they cause and the surgical treatments required are often very expensive [2]. Moreover, there is a high level of risk associated among women with the disease, particularly those over the age of 40 years, with the risk peaking in the fourth and fifth decades of life [3].

The three main types of gallstones are pigment, cholesterol, and mixed stones [4]. In both Asian and Western populations, cholesterol stones represent the most prevalent type. Crystallization, reduced gallbladder motility, and supersaturation of bile with cholesterol are the typical causes of stone formation [5]. Many patients remain asymptomatic and are diagnosed incidentally during imaging studies or surgical procedures, whereas symptomatic patients may exhibit biliary colic, nausea, vomiting, or dyspepsia [6].

Cholelithiasis is associated with risk factors such as age, sex, parity, ethnicity, family history, and dietary composition [7]. Obesity and a higher body mass index (BMI) are significant risk factors as they enhance hepatic cholesterol synthesis, which leads to lithogenic bile. There is still a lack of regional data from Pakistan, although a study from southern Sindh reported that the prevalence was 4% among men and 14.2% among women [8]. This study aimed to develop management and prevention strategies by understanding the risk factors and prevalence of cholesterol gallstones, particularly obesity. In a regional context, addressing this knowledge gap can help assess the association between high BMI and the development of cholesterol stones [9].

Thus, the objectives were to determine the prevalence of cholesterol gallstones in cholelithiasis patients, evaluate the relationship between obesity and cholesterol stones, and identify additional risk factors. This study also aimed to suggest region-specific preventive measures and identify the common sites in the gallbladder where cholesterol stones tend to lodge due to impaired cholesterol metabolism.

## Materials and methods

This descriptive, cross-sectional study was conducted from March to August 2025 in the Department of Surgery at Jinnah Medical and Dental College and affiliated hospitals, including Sohail Trust Hospital in Karachi. The study was approved by the Institutional Ethics Committee (approval number: JMDC/00110/25). Patients aged above 18 years with cholelithiasis confirmed through ultrasonography and admitted for medical or surgical treatment were included in the study. Patients with a history of biliary surgery, chronic liver disease, hemolytic disorders, acute cholecystitis, or choledocholithiasis were excluded. Before data collection, each participant provided written informed consent. A total of 300 patients were included through a non-probability purposive sampling technique. OpenEpi version 3.0 (Atlanta, GA, USA) was used to determine the minimum sample size with a 95% confidence level, 5% margin of error, and an expected prevalence of 70% based on prior regional data [10,11].

A structured questionnaire and clinical proforma were used to collect data based on past research on the risk factors of gallstones [12]. The tool was also pretested to be understandable and reliable. Anthropometric measurements were obtained using calibrated digital weighing scales and stadiometers. All tests were conducted by qualified personnel under surgical supervision with ordinary hospital equipment that did not require a license. The questionnaire contained demographic data (age, sex, marital status, and occupation), comorbidities (diabetes, hypertension, hepatitis, tuberculosis, and depression), family history of obesity or gallstones, and lifestyle choices (diet, exercise, smoking, and meal frequency).

The data on dietary and lifestyle factors were obtained using a structured questionnaire on the type and frequency of fatty food consumption, physical activity (sedentary, moderate, or active), number of meals per day, and smoking history (present/absent). The frequency and the predominant composition of food intake per day by the respondents categorized the dietary pattern. A fatty diet consisted of the consumption of fried foods, red meat, butter, ghee, cream, or processed snacks at least four times per week, or more than half the meals were rich in visible fats or oils. A balanced diet meant consuming both vegetarian and non-vegetarian foods with moderation of fats (two to three times per week) and incorporation of vegetables, pulses, and grains in routine food. A non-fatty or balanced diet consisted of mainly boiled, grilled, or baked food with a minor amount of visible fat (fewer than two times a week).

These were considered as adjustable risk factors in the development of gallstones. The main variable was the type of gallstones (cholesterol, mixed, pigment) determined intraoperatively by the conventional morphological characteristics (color, texture, and sectioning). Cases that lacked calculi but showed ultrasonographic signs of the presence of sludge or microlithiasis were regarded as acalculous cholelithiasis. BMI was the key independent variable (calculated weight (kg)/height (m²) and classified using the World Health Organization (WHO, 2022) criteria as follows [13]: underweight (<18.5 kg/m²), normal (18.5 -24.9 kg/m²), overweight (25.0-29.9 kg/m²), obese class I (30.0-34.9 kg/m²), obese class II (35.0-39.9 kg/m²), and obese class III (40.0 kg/m² and above).

Data analysis was performed using SPSS Statistics version 26.0 (IBM Corp., Armonk, NY, USA). Continuous variables were represented as mean and standard deviation (SD), and categorical variables as frequencies and percentages. An independent t-test was used to compare the means between the sexes, and the chi-square test was used to determine the relationship between the BMI categories and the type of gallstones and other categorical variables. The statistically significant p-value was assumed at 0.05. All formulas and instruments applied in this research (BMI classification, SPSS software, and anthropometric devices) were either free of charge or licensed at the institution and did not require further permissions.

## Results

This study included 300 patients who had cholelithiasis confirmed by ultrasonography. The participants’ mean age was 41.2 ± 11.5 years, and a significant majority were female (n = 261, 87%). The preoperative mean BMI was 28.5 ± 5.4 kg/m² (range = 18.0 to 42.0 kg/m²). Table 1 provides a summary of the study population’s anthropometric and demographic features.

**Table 1:** Demographic and anthropometric characteristics of the study population (n = 300). *: Significant at p-values <0.05. BMI: body mass index; SD: standard deviation; NS: not significant; df: degree of freedom

Parameter	Total (N = 300), n (%)	Male (N = 39), n (%)	Female (N = 261), n (%)	Statistical test	Test value	P-value
Age categories						
<30 years	79 (26.3)	11 (28.2)	68 (26.1)	Chi-square	44.46^df=4^	0.001*
31–40 years	101 (33.7)	28 (71.8)	73 (28.0)			
41–50 years	79 (26.3)	26 (66.7)	53 (20.3)			
51–60 years	35 (11.7)	2 (5.1)	33 (12.6)			
>60 years	6 (2.0)	0 (0.0)	6 (2.3)			
Occupation						
Stay at home	253 (84.3)	11 (28.2)	242 (92.7)	Chi-square	16.68^df=1^	0.005*
Professional worker	47 (15.7)	28 (71.8)	19 (7.3)			
Marital status						
Single	22 (7.3)	2 (5.1)	20 (7.7)	Chi-square	80.25^df=2^	<0.001*
Married	274 (91.3)	35 (89.7)	239 (91.6)			
Divorced	4 (1.3)	2 (5.1)	2 (0.8)			
Height (m), mean ± SD	1.562 ± 0.07	1.59 ± 0.06	1.56 ± 0.07	t-test	t = 1.20	0.231
Weight (kg), mean ± SD	68.39 ± 12.0	73.2 ± 13.2	67.5 ± 11.8	t-test	t = 1.69	0.092
Preoperative BMI (kg/m²), mean ± SD	28.5 ± 5.4	27.2 ± 4.8	28.8 ± 5.6	t-test	t = 1.32	0.188

The prevalence of being overweight or obese was significantly higher in females than in males (p < 0.001). The distribution of age, occupation, and marital status also showed significant differences between the sexes. To assess patterns of stone composition, postoperative gallstone types were compared with preoperative BMI. Chi-square tests were used to examine associations. The data are presented in Table 2.

**Table 2:** Postoperative gallstone type by preoperative BMI categories (n = 300). *: Significant at p-values <0.001. BMI: body mass index; df: degree of freedom

BMI category	Total, N (%)	Cholesterol, N (%)	Mixed, N (%)	Pigmented, N (%)	Acalculous, N (%)	Statistical value	Test value	P-value
Underweight (<18.5 kg/m²)	9 (3.0)	0 (0.0)	5 (55.6)	4 (44.4)	0 (0.0)	Chi-square	127.449^df=20^	0.000*
Normal (18.5–24.9 kg/m²)	81 (27.0)	14 (17.3)	22 (27.2)	26 (32.1)	19 (23.5)			
Overweight (25–29.9 kg/m²)	101 (33.7)	63 (62.4)	29 (28.7)	9 (8.9)	0 (0.0)			
Obese Class I (30–34.9 kg/m²)	86 (28.7)	66 (76.7)	15 (17.4)	4 (4.7)	1 (1.2)			
Obese Class II (35–39.9 kg/m²)	19 (6.3)	17 (89.5)	0 (0.0)	0 (0.0)	2 (10.5)			
Obese Class III (≥40 kg/m²)	4 (1.3)	2 (50.0)	2 (50.0)	0 (0.0)	0 (0.0)			

While cholesterol stones were more common in overweight and obese patients, mixed and pigmented stones were more common in normal and underweight patients. To determine the trends related to demographic and anthropometric factors, the presenting complaints, related comorbidities, and familial predispositions were examined. t-tests were employed for continuous measures, and chi-square tests for categorical variables. Details are displayed in Table 3.

**Table 3:** Clinical and comorbidity characteristics of the study population (n = 300). *: Significant at p-values <0.05. df: degree of freedom

Parameter	Total (N = 300), n (%)	Male (N = 39), n (%)	Female (n = 261), n (%)	Statistical test	Test value	P-value
Presenting complaint						
Pain in the epigastrium	13 (4.3)	0 (0.0)	13 (5.0)	Chi-square	17.552^df=3^	0.287
Pain in the right hypochondrium	280 (93.3)	39 (100)	241 (92.3)			
Pain in the epigastrium and right hypochondrium	6 (2.0)	0 (0.0)	6 (2.3)			
Pain in the right hypochondrium and back	1 (0.3%)	0 (0.0)	1 (0.4)			
Associated comorbidities						
None	228 (76.0)	9 (23.1)	219 (83.9)	Chi-square	202.872^df=6^	0.000*
Depression	2 (0.7)	0 (0.0)	2 (0.8)			
Diabetes mellitus	20 (6.7)	4 (10.3)	16 (6.1)			
Hypertension	31 (10.3)	5 (12.8)	26 (10.0)			
Diabetes + hypertension	12 (4.0)	2 (5.1)	10 (3.8)			
Hepatitis C	3 (1.0)	0 (0.0)	3 (1.1)			
Hepatitis B and C	2 (0.7)	0 (0.0)	2 (0.8)			
Tuberculosis	2 (0.7)	0 (0.0)	2 (0.8)			
Family history of cholelithiasis	82 (27.3)	22 (56.4)	60 (23.0)	Chi-square	11.222^df=1^	0.047*
Familial obesity	94 (31.3)	0 (0.0)	94 (36.0)	Chi-square	59.07^df=1^	0.000*

The most common presenting complaint was pain in the right hypochondrium. However, higher BMI was substantially correlated with a family history of cholelithiasis and familial obesity (p = 0.047 and p < 0.001, respectively). To determine their possible impact on gallstone formation, dietary and lifestyle choices were examined. Chi-square tests were used to examine associations with BMI. The findings are displayed in Table 4.

**Table 4:** Lifestyle and dietary characteristics of the study population (n = 300). *: Significant at p-values <0.05. df: degree of freedom

Parameter	Total (N = 300), n (%)	Statistical test	Test value	P-value
Type of diet				
Fatty diet	87 (29.0)	Chi-square	34.655^df=1^	0.000*
Mixed food	213 (71.0)			
Frequency of fatty food intake				
Rarely (<1/week)	31 (10.3)	Chi-square	127.160^df=3^	0.000*
Occasional (1–2/week)	90 (30.0)			
Moderate (3–4/week)	130 (43.3)			
Frequent (5–6/week)	49 (16.3)			
Exercise				
Sedentary/Light	165 (55.0)	Chi-square	61.361^df=2^	0.000*
Active/Moderate	124 (41.3)			
Highly active	11 (3.7)			
Smoking history				
Absent	278 (92.7)	Chi-square	6.558^df=1^	0.256
Present	22 (7.3)			
Number of meals/day				
1–2	75 (25.0)	Chi-square	45.010^df=3^	0.001*
3	196 (65.3)			
4	22 (7.3)			
>4	7 (2.3)			

The majority of patients had a varied diet, including moderate amounts of fatty foods. There were no notable differences in smoking history or exercise levels between BMI categories (p = 0.256). BMI and the number of meals consumed each day were found to be significantly correlated (p = 0.001).

## Discussion

This study assessed the relationship between BMI and the type of gallstones in adult patients with cholelithiasis. According to this study, cholesterol gallstones accounted for 54.0% (n = 162) of calculi among patients with cholelithiasis, with mixed (n = 73, 24.3%) and pigmented stones (n = 43, 14.3%). The prevalence of cholesterol gallstones increased gradually from 14 (17.3%) in the normal BMI group to 17 (89.5%) in the obese class II group, indicating a significant correlation between BMI and cholesterol gallstone formation. Cholesterol stones were also prevalent in patients who were overweight and in the obese class I group (62.4%, n = 63, and 76.7%, n = 66, respectively). These results confirm that elevated BMI is a strong predictor of the formation of cholesterol gallstones. They are also consistent with other studies that found obesity to be a major determinant of cholesterol supersaturation and gallstone pathogenesis [14,15].

In this cohort, women comprised 87% (n = 261) of the study population, indicating a clear female predominance. Compared with men, women had a higher prevalence of being overweight and obese, as well as a higher mean BMI. This aligns with the body of research showing that female gender is a significant risk factor for cholelithiasis, most likely as a result of hormonal influences [16]. Both estrogen and progesterone may decrease gallbladder motility and increase hepatic cholesterol secretion, which together contribute to lithogenic bile. Obese patients might have a higher incidence of cholesterol stones because of impaired bile stasis and emptying of the gallbladder, which could promote the formation and growth of cholesterol crystals. The identification of these patterns was found to be clinically significant because they could impact surgical planning, predict possible intraoperative difficulties, and guide postoperative follow-up strategies, especially in populations with high obesity rates [17,18].

It was biologically plausible that obesity and cholesterol gallstones are strongly related [19]. Overeating decreases the production of bile acids, increases the production of hepatic cholesterol, and impairs the contractility of the gallbladder. Insulin resistance linked to obesity can also worsen bile stasis and produce a lithogenic environment. In addition, several earlier studies have shown a dose-response relationship between gallstone formation and BMI, highlighting obesity as a modifiable risk factor [20].

This study had some limitations. As the design was cross-sectional, it could not establish a causal relationship between the development of cholesterol gallstones and BMI. Accuracy might have been impacted because stone classification was based on visual morphological evaluation rather than biochemical analysis. Despite these limitations, the study highlighted the role BMI plays in the development of cholesterol gallstones. Future studies should adopt a longitudinal design with biochemical stone analysis so that causal relationships can be established and the impact of targeted weight reduction interventions on gallstone prevention can be assessed.

## Conclusions

Most of the gallstones noted in the study were cholesterol gallstones, which had a strong positive correlation in regard to their development and higher BMI. Overweight and obese patients had a higher proportion of cholesterol stones, which demonstrated that obesity is a major modifiable risk factor. Female patients were disproportionately affected due to their higher BMI and likely hormonal influences. These results underscore the importance of controlling cholesterol, possibly through dietary improvements and weight reduction, in the prevention of gallstones. The region-specific data of this study can aid in the creation of clinical management plans and focused community health policies. Other metabolic, hereditary, and behavioral factors that may be involved in gallstone formation should be the focus of subsequent research.
